# Trait Mindfulness as a Limiting Factor for Residual Depressive Symptoms: An Explorative Study Using Quantile Regression

**DOI:** 10.1371/journal.pone.0100022

**Published:** 2014-07-02

**Authors:** Sholto Radford, Catrin Eames, Kate Brennan, Gwladys Lambert, Catherine Crane, J. Mark G. Williams, Danielle S. Duggan, Thorsten Barnhofer

**Affiliations:** 1 Centre for Mindfulness Research and Practice, Bangor University, Dean St Building, Bangor, United Kingdom; 2 Department of Psychiatry, University of Oxford, Warneford Hospital, Oxford, United Kingdom; 3 School of Ocean Sciences, Bangor University, Menai Bridge, United Kingdom; Charité-Universitätsmedizin Berlin, Germany

## Abstract

Mindfulness has been suggested to be an important protective factor for emotional health. However, this effect might vary with regard to context. This study applied a novel statistical approach, quantile regression, in order to investigate the relation between trait mindfulness and residual depressive symptoms in individuals with a history of recurrent depression, while taking into account symptom severity and number of episodes as contextual factors. Rather than fitting to a single indicator of central tendency, quantile regression allows exploration of relations across the entire range of the response variable. Analysis of self-report data from 274 participants with a history of three or more previous episodes of depression showed that relatively higher levels of mindfulness were associated with relatively lower levels of residual depressive symptoms. This relationship was most pronounced near the upper end of the response distribution and moderated by the number of previous episodes of depression at the higher quantiles. The findings suggest that with lower levels of mindfulness, residual symptoms are less constrained and more likely to be influenced by other factors. Further, the limiting effect of mindfulness on residual symptoms is most salient in those with higher numbers of episodes.

## Introduction

Depression is a serious human health problem; in terms of burden of disease the World Health Organisation ranks it at first place in middle- and high-income countries and at third place worldwide [1]. Major Depression diagnoses have increased considerably in recent decades [2] and for many sufferers, depressive episodes are not isolated events. Relapse is very common, and for a considerable proportion of those affected depression can become a chronic/recurrent condition.

Important risk factors for recurrence of major depression include the number of previous episodes and residual symptomatology [3]. From a psychological perspective, it has been argued that both of these factors increase the likelihood that learned maladaptive patterns of depressogenic thinking become reactivated. The differential activation account of recurrent depression suggests that during episodes of depression associations are formed between low mood, hopelessness, worthlessness and other symptoms or characteristics of depression, and that these associations are strengthened over repeated incidences of negative mood. During times of remission, these learned patterns remain latent [4], [5]), but can be easily re-activated through only subtle changes in mood, a phenomenon that is referred to as cognitive reactivity [6], [7], [8]. Cognitive reactivity has been shown to be a significant predictor of relapse, and is likely to increase with number of previous episodes [7].

The differential activation account implies that residual symptoms may play an important role in risk for relapse. Consensus definitions of remission allow for a minimum level of symptoms to be present, and research has found that residual symptoms are a frequent occurrence. In line with the above suggestion, research has shown that compared with those who are asymptomatic, individuals with residual symptoms have a significantly higher risk of relapse [9], [10]. Residual symptoms have therefore increasingly become a target for treatment in their own right [11], [12], [13], [14].

Training in mindfulness meditation, a practice that enhances individuals' capacities to orient their attention and awareness to present moment experience, and to respond to their experience with acceptance and non-judgement, rather than rumination and elaboration, seems to offer particular benefits to patients with recurrent depression. Such an orientation is considered to be orthogonal to the maladaptive habitual patterns of thinking that patients with recurrent depression often engage in, and consequently has been assumed to be an important protective factor [15]. Consistent with this, a number of clinical trials have now demonstrated that Mindfulness-Based Cognitive Therapy (MBCT), an intervention combining intensive training in mindfulness meditation and elements from cognitive therapy, effectively reduces risk for relapse in patients with recurrent depression (for an overview see the recent meta-analysis by Piet & Hougaard [16]). However, preventative effects seem to be restricted to a particular group of patients, i.e. the training has been found to be effective for patients with more than three previous episodes but not for patients with one or two previous episodes, and within this group particularly for patients who do not reach stable recoveries, i.e. those who continue to show residual symptoms [17]. While mindfulness is assumed to be a general protective factor, the above findings suggest that its effects in patients suffering from recurrent depression may vary depending on contextual factors such as number of previous episodes and level of residual symptoms. Further investigation of these effects may help to elucidate the mechanisms of action of mindfulness training.

In the current study, we investigated effects of trait mindfulness in patients with recurrent depression. Increasing attention has been paid in recent years to measuring mindfulness, and alongside this development has been the emerging theme that mindfulness can be described as a trait. That is, that mindfulness can be considered in terms of dispositional individual differences that can be assessed at the trait level, as well as being considered a state-level construct [18], [19], [20], [21]. Trait mindfulness seems to be negatively associated with psychological distress and rumination [18], [22], [23], [24], [25], and positively associated with life satisfaction [18], empathy [26], sense of autonomy, and pleasant affect [18]. Here we were interested in exploring the relation between mindfulness and depressive symptoms in a more detailed way by taking into account the contextual factors that clinical trials have identified as crucial moderating variables, namely the number of previous episodes and the severity of residual symptoms. While we assumed that overall there is a negative relation between mindfulness and symptoms of depression, i.e. that higher levels of mindfulness are associated with lower levels of depression, our goal was to explore whether the strength of this relation differed depending on the above contextual factors.

It is hypothesised that residual symptoms represent low level manifestations of depressive processes, and that the ability to be aware of these processes (and in turn to bring an orientation of present focused non-judgement towards them), may prevent them from both persisting as residual symptoms and from worsening into full major depressive episodes. Mindfulness-based interventions such as MBCT [27] specifically aim to cultivate mindfulness, and there is indeed evidence for levels of trait mindfulness to significantly increase following training [28]. If trait mindfulness operates in a similar way to mindfulness developed through intervention, then levels of trait mindfulness may act to limit the level of residual symptoms between depressive episodes. Although there is a reasonable rationale to support the suggestion that having high levels of trait mindfulness should be protective against depressive symptoms (due to the accumulating evidence of the association between mindfulness and adaptive psychological functioning), theories of depression tell us that the risk factors associated with depression are complex and multifaceted involving an interaction of biological, social, and psychological factors [29]. It does not necessarily hold therefore, that individuals with low trait mindfulness will always have a high level of symptoms. Rather, given the likely complexity of factors influencing depressive symptoms, it is more plausible to assume that trait mindfulness *may act as a limiting factor* for depressive symptomology. Specifically we propose that at low levels of trait mindfulness, many factors will determine the presence or absence of depressive symptoms and mindfulness will have comparably little impact on these processes. However, at high levels of trait mindfulness residual symptoms will be limited because, irrespective of what is triggering them, high trait mindfulness will prevent them from being maintained or exacerbated. Furthermore, it is conceivable that this limiting effect of trait mindfulness is more pronounced for those individuals in whom residual symptoms can be triggered more easily by differential activation processes, i.e. individuals with higher numbers of prior episodes of depression, since mindfulness is proposed to act on these psychological processes to limit symptom escalation. We would thus expect the number of previous episodes to act as a moderating variable.

The concept of limiting factors has been extensively explored within ecology [30] using quantile regression. Quantile regressions can be employed where problems of unequal variances arise, and have the advantage over non-parametic regression models such as polynomial regression, Spline regression and LOESS regression, in that they are not constrained to predictions of central tendency. Indeed, relations can often be fitted to other parts of the distribution and the mean may not always be the most meaningful response variable. Ecologists argue that, when there are potentially many factors constraining the response variable of the model and when all those factors have not been measured, i.e. when there are many hidden limiting factors, the effect of the predictor of interest will be greater in the upper limit of the distribution, i.e. upper quantiles. This is because, if the predictor has an effect on the response variable, the highest values in the distribution of the response along the predictor gradient will be constrained by the predictor itself whereas all the remaining lower values, scattered underneath, will be limited by a combination of hidden factors [30], [31].

To our knowledge the concept of limiting factors, and the associated statistical procedures for their testing, has not been applied when investigating the relationship between psychological processes, but may add value for a number of reasons. First, in psychological research it is not always possible to get accurate measures of all the potential psychological and situational factors that may contribute towards an outcome measure such as depressive symptoms. In this case when modelling a predictor-outcome relationship, issues of heteroscadecity may arise, particularly if there are different rates of change in the outcome variable associated with unmeasured factors. Unlike standard least squared linear regression models, quantile regression is not based on the assumption of homoscedasticity and can be used to compute slope estimations throughout the distribution giving a fuller, more nuanced, picture of the whole distribution. Second, having a slope estimation for the predictor of interest at the higher end of the distribution (where it is acting as the principle limiting factor) may be useful in drawing inferences which are clinically meaningful. For example, it gives an indication of the level of the predictor required to limit the outcome to a certain level, while taking into account all unmeasured variables (i.e. what level of trait mindfulness might need to be cultivated to have clinically meaningful effects). Standard linear models based around the mean are unable to give estimates at noncentral locations in this manner.

In the current study we aimed to explore whether trait mindfulness acts as a limiting factor in determining the level of residual depressive symptoms reported by individuals who have a history of recurrent major depression but are currently out of episode. In the current context, we defined residual symptoms as the self-reported levels of symptoms on the Beck Depression Inventory II [32] in patients who had previously fulfilled our inclusion criteria for remission as assessed by structured clinical interview. As the structured interview and self-reports were assessed at two separate consecutive points of assessment about a week apart, it is possible for self-reports of symptoms to be affected by deteriorations in condition between assessment points and for average scores therefore to be slightly higher than the levels of symptoms that consensus definitions refer to as residual symptoms, i.e. levels of symptoms below a score of 7 on the HAMD rating scale. We hypothesised (a) that the natural ability to be aware of and act in a less judgmental way towards low level residual symptoms (trait mindfulness) would limit symptom escalation, particularly at high levels of trait mindfulness where the influence of mindfulness is likely to be more dominant among the range of influencing factors, and (b) that such limiting would be more evident among those individuals for whom automatic and habitual reactivation of depressogenic cognitions is hypothesised to be the dominant mechanism of relapse, i.e. those individuals with a greater number of prior depressive episodes.

## Methods

### Participants

The current sample consisted of participants who were recruited to take part in the Staying Well after Depression Trial – a randomized-controlled trial comparing MBCT and a psychological control treatment (Cognitive Psycho-Education), to usual care in the prevention of relapse to depression (*N* = 274; see Williams et al. [33], for full trial protocol). Main inclusion criteria were (a) a recent history of recurrent major depression, defined as at least three episodes of depression in the past, two of which occurred in the past five years and one within the past two years, (b) meeting criteria for recovery from depression as defined by the National Institute for Mental Health (NIMH) guidelines, i.e. potential trial participants were deemed *not* to be in recovery or remission, and hence *ineligible*, if they reported that at least one week during the previous 8 they had experienced *either* a core symptom of depression (depressed mood, anhedonia) *or* suicidal feelings plus at least one other symptom of depression, (c) age between 18 and 70 years of age. Participants had to be without a language difficulty or visual impairment that would prevent them from completing the research assessments. Those reporting regular self-harm or current suicidal ideation were also excluded, as were potential participants with a history of schizophrenia, schizoaffective or bipolar disorder along with those with a current diagnosis of an obsessive-compulsive disorder or an eating disorder.

The sample comprised of 72.3% women (*n* = 198) and 27.7% men (*n* = 76) with a mean age of 43.93 years (18–68). With regards to current socioeconomic and employment status, 101 (37.4%) were currently in full-time employment and 54 (19.7%) were working on a part-time basis. The majority of participants (76.6%) identified themselves as White-British. All the participants were fluent in English and 88.3% (*n* = 242) of the sample identified English as their first language. Of the 274 participants who provided data for the current analyses, 120 (43%) were currently taking antidepressants. In line with exclusion criteria, none of the participants were currently receiving individual psychotherapeutic treatment; 78 (28%) reported engaging in some form of guided self-help. Structured clinical interviews showed that 103 (38%) had a current diagnosis or a history of anxiety disorder, and 43 (16%) had a history of a substance-related disorder. The trial was multi-centred and recruitment took place across two sites, at Oxford and Bangor Universities, UK. Participants were recruited from the community through poster and media advertisements. General Practitioners (GPs) were informed about the study and invited to provide any potentially suitable patients with the contact details of the local recruitment team. Having contacted the recruitment team, potential participants were screened over the phone for the principal inclusion and exclusion criteria. Those who appeared to be eligible on the basis of telephone screening were invited for a more detailed assessment of suitability, conducted by a trained researcher. Diagnostic eligibility and lifetime clinical history was assessed using the Structured Clinical Interview for DSM-IV [34] and followed up with self-report measures of mood and cognitive factors, including the Beck Depression Inventory and the Five-Factor Mindfulness Scale used in this study.

### Ethics Statement

The Oxfordshire Research Ethics Committee C and the North Wales Research Ethics Committee gave approval for the study in July 2008. All subjects provided written informed consent prior to their participation in the study.

### Measures

#### Structured Clinical Interview for DSM-IV (SCID [34])

The SCID, comprising all components of the interview, was conducted by trained research assistants to assess current diagnostic status and history of major depressive disorder. Research assistants were extensively trained using the sequence of steps suggested by the developers of the interview including the use of case studies on DVD. Studies investigating inter-rater reliability of the SCID have consistently yielded Kappa values above .60 for most categories (see for example [35]).

#### Beck Depression Inventory (BDI-II [32])

The BDI-II is a well-established 21-item self-report questionnaire used to measure severity of current symptoms of depression. The twenty-one items assess symptoms over the preceding two weeks. There is a maximum score of 63 on the total questionnaire with four cut-off ranges indicating minimal (scores between 0–13), mild (14–19), moderate (20–28) and severe (29–63) symptoms of depression [27]. A higher score on the scale indicates a greater severity of depression. Internal consistency in the current sample was α = .90.

#### Five-Facet Mindfulness Questionnaire (FFMQ [36])

The Five-Facet Mindfulness Questionnaire is a 39-item questionnaire used to measure dispositional mindfulness, or the general tendency to be mindful in daily life. The factors that make up this scale were drawn from an exploratory factor analysis of several measures of mindfulness that suggested a five-factor solution, all of which are components of an overall mindfulness construct [36], [37]. The five factors include: Observing: noticing or attending to internal and external experiences; Describing: labelling internal experiences with words; Acting with awareness: focusing on one's activities of the moment rather than placing attention elsewhere; Nonjudging of inner experience: taking a non-evaluative stance toward thoughts and feelings, and Nonreactivitiy to inner experience: the tendency to allow thoughts and feelings to come and go without getting caught up in them. The five facets demonstrate adequate to good internal consistency, with alpha coefficients ranging from .75 to .91 [37]; internal consistency for the overall scale in the current sample was α = .90.

### Data Analysis

To investigate the relationship between levels of trait mindfulness and residual depressive symptoms, we used quantile linear regressions [38]. As described above, we chose this method as it is suitable for data with unequal variances and unlike other non parametric regression models it allows for the examination of limiting factors. Quantile regressions split the data into quantile classes, thus allowing examination of the predictor response relationship throughout the distribution. For instance, the model fitted at the 95th quantile describes the function for which 95% of the observations fall under the fitted line. The 50^th^ quantile represents the function for which 50% of the observations fall beneath the fitted line and thus represents the median. The function fits a model by minimising a weighed sum of absolute residuals and the significance of the relationship is assessed by standard resampling methods, i.e. bootstrapping [39], [40].

This method was of particular interest in our study as our sample consisted of individuals who are currently in remission from depression but with varying degrees of residual symptoms. Although we predicted trait mindfulness would be a key limiting factor we also expected that a number of other unmeasured social, biological, and psychological variables would also be affecting levels of residual symptoms. Unlike standard linear regression, quantile regression does not assume homoscedasticity of variance and indeed observation of a residuals vs. fitted values plot indicated heteroscedasticity within our data.

All analysis were conducted using R.

## Results

### Trait mindfulness as a limiting factor of residual depressive symptoms

**Figure 1:**
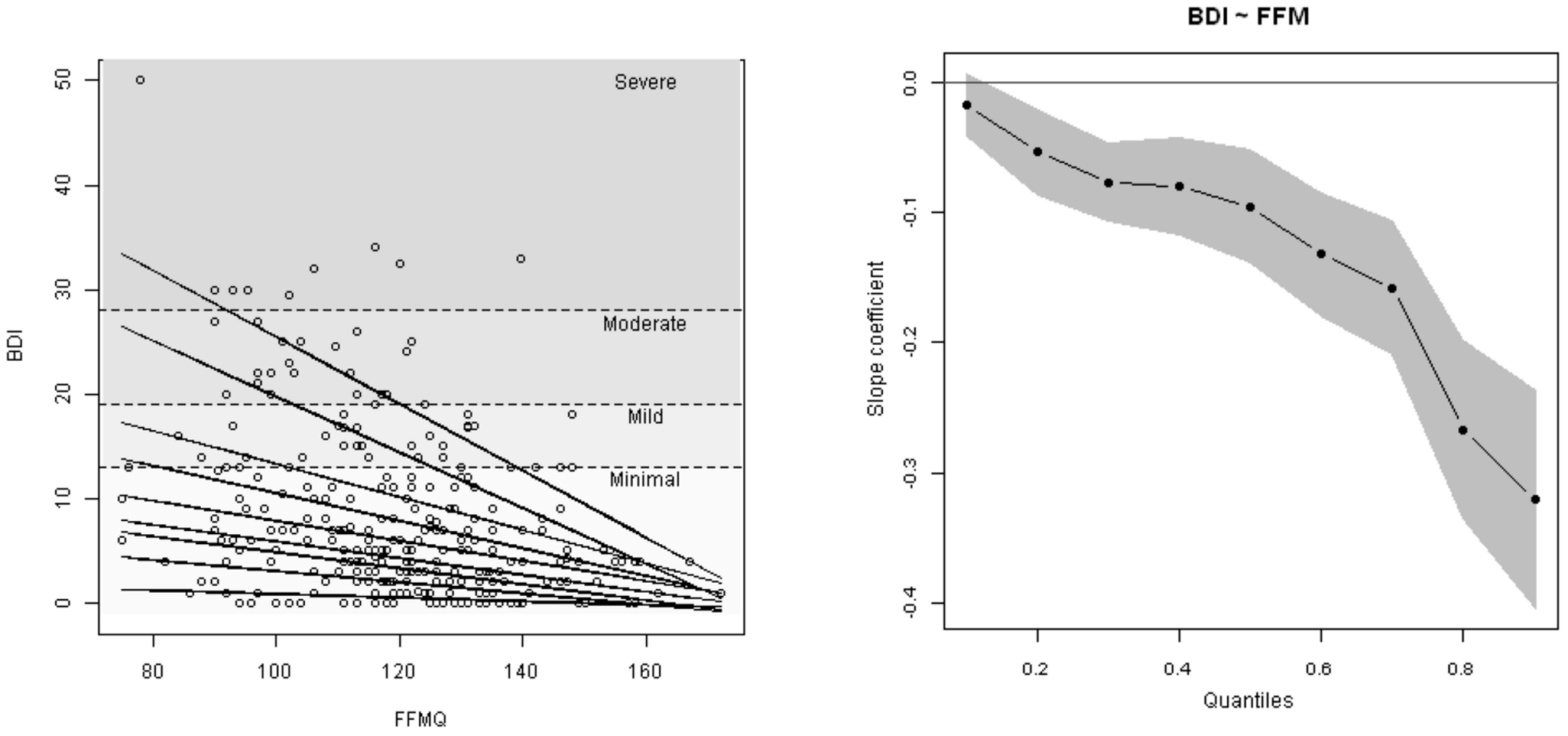
Quantile regression of BDI as a function of FFMQ. (1A) Scatter plot with model predictions at the different quantiles (from top to bottom 0.9,0.8,0.7,0.6,0.5,0.4,0.3,0.2,0.1.). Grey shaded areas with dotted dividing lines represent symptom severity categories from BDI cut-off. (1B) Slope coefficient estimates at the different quantiles and their confidence intervals. Grey Band represents 90% confidence interval around the slope estimation.

Results of the quantile regression indicate that significant negative relationships exist between FFMQ (Five-Facet Mindfulness Questionnaire) and BDI (Beck Depression Inventory) across the distribution with the exception of the 10th quantile (see Table 1 and Figure 1). Figure 1A shows the relationship between FFMQ and BDI at different quantiles while Figure 1B illustrates the increase in steepness and significance of the slopes between the 10^th^ and 90^th^ quantiles. Only at the 10^th^ quantile does the confidence interval include 0 indicating that the slope is not significant. Overlapping confidence intervals between the 20th and 70^th^ quantiles (Figure 1B) indicate no significant difference in slope steepness. However, there is significant change in slope steepness between the 70^th^ and 90^th^ quantiles demonstrated by lack of overlap in confidence intervals. This suggests that FFMQ acts as a limiting factor of BDI with rates of change increasing at the quantiles near the maximum response and that usual regression methods would fail to capture the steepness of this relationship due to the existence of other unmeasured factors which influence BDI. As well as using the total FFMQ score we examined the five subscales of the FFMQ using quantile regression throughout the response distribution. Similar trends were evident for each subscale and the total score. To examine if any of the subscales individually gave a better account for BDI than the total FFMQ score, we examined Akaike Information Criterion (AIC) values at the upper quantiles (.75, .80, .85 and .90). In all cases AIC values for total score were lower than for any of the individual subscales, indicating that the model using the total score offered the best fit.

**Table 1:** Quantile Regression of FFMQ Total Score on BDI-II Total Score at a Range of Quantiles Through the Response Distribution.

Quantile	Slope	*SE*	*t*	*p*	*R1*
0.1	−0.01	0.01	−1.12	.26	0.02
0.2	−0.05	0.02	−2.42	.01	0.03
0.3	−0.07	0.01	−4.23	.00	0.04
0.4	−0.08	0.02	−4.06	.00	0.05
0.5	−0.09	0.02	−3.70	.00	0.05
0.36	−0.13	0.03	−4.47	.00	0.06
0.7	−0.15	0.03	−5.25	.00	0.08
0.8	−0.26	0.04	−6.71	.00	0.10
0.9	−0.32	0.05	−6.36	.00	0.14

*Note*. R1 is a pseudo-R^2^ value that reflects the local goodness of fit around a particular quantile [54]. The estimate is based on the comparison of the weighed residuals of a restricted versus unrestricted model with residuals weighed depending on their sign (if negative they are multiplied by (tau – 1), tau being the quantile of interest, if positive they are weighed by tau).

### Previous number of episodes as a moderator

Given the association between risk of relapse and both number of previous episodes of depression and residual symptomology, we investigated whether individuals who had a greater number of previous episodes (and hence it is assumed a more established pattern of depressive processing) differed from those with fewer prior episodes in how important trait mindfulness was as a factor limiting residual symptoms while out of episode. A considerable proportion of the sample (*n* = 192) had experienced between three and eight episodes but a number of individuals (*n* = 48) reported more than 8 episodes ranging as high as 45, or had experienced too many episodes to give an exact figure (*n* = 34). Due to non-parametric distribution, and the absence of true values within the higher range (*n* = 34) we used previous episodes as a categorical rather than continuous variable. In order to capture this range in a meaningful way, as well as allowing for a reasonably even split between categories, we coded individuals with 3–4 episodes in the lower category (A; *n* = 104), 5–8 in the medium category (B; *n* = 88) and >8 in the higher category (C; *n* = 82). The 34 participants who had experienced too many episodes to give estimation are included in category C. One-way ANOVAs were conducted to establish whether these categories differed in respect to demographic and clinical characteristics (see Table 2). Categories differed significantly in terms of their current age, age of onset of first episode, and total FFMQ score. Tukey post-hoc comparisons revealed that mean age of category A, *M* = 41.69, 95% CI [39.11, 44.27], was significantly lower than category C, *M* = 46.45, 95% CI [43.96, 48.93]. Age of onset for category A, *M = 24.10*, 95% CI [21.79, 26.40], was significantly later than category B, *M* = 18.98, 95% CI [17.02, 20.93], and category C, *M* = 19.11, 95% CI [16.91, 21.31]. Total FFMQ for Category B, *M* = 123.62, 95% CI [120.03, 127.21], was significantly higher than for category C, *M* = 114.98, 95% CI [111.54, 118.42].

**Table 2:** Demographic Characteristics and Group Differences based on Previous Number of Major Depressive Episodes.

	Total sample (n = 274)	Previous number of depressive episodes			Analysis		
Characteristic		3–4 (n = 103)	5–8 (n = 88)	>8 (n = 82)	*F*	*df*	*p*
Age *M* (*SD*)	43.93 (12.02)	41.69 (13.25)	44.21 (10.68)	46.45 (11.32)	3.69	2	0.02
Gender % female	72.3	72.1	77.3	67.1	1.10	2	0.33
ADs last 7 days % yes	48.6	47.4	54.4	43.7	0.91	2	0.40
Previous CBT % yes	20.3	16.1	23.8	28.6	0.38	2	0.68
Age of onset *M* (*SD*)	20.95 (10.73)	24.01 (11.79)	18.98 (9.23)	19.11 (10.0)	7.46	2	0.00
BDI-II *M* (*SD*)	8.25 (8.11)	7.37 (6.75)	8.08 (7.62)	9.55 (9.92)	1.70	2	0.18
FFMQ *M* (*SD*)	119.05 (17.94)	118.35 (19.68)	123.62 (16.95)	114.98 (15.56)	5.183	2	0.00

*Note*. FFMQ = Five Facet Mindfulness Questionnaire total score, BDI-II = Beck Depression Inventory – II total score, ADs = antidepressant medication. Percentage values refer to percentage of valid responses.

**Figure 2:**
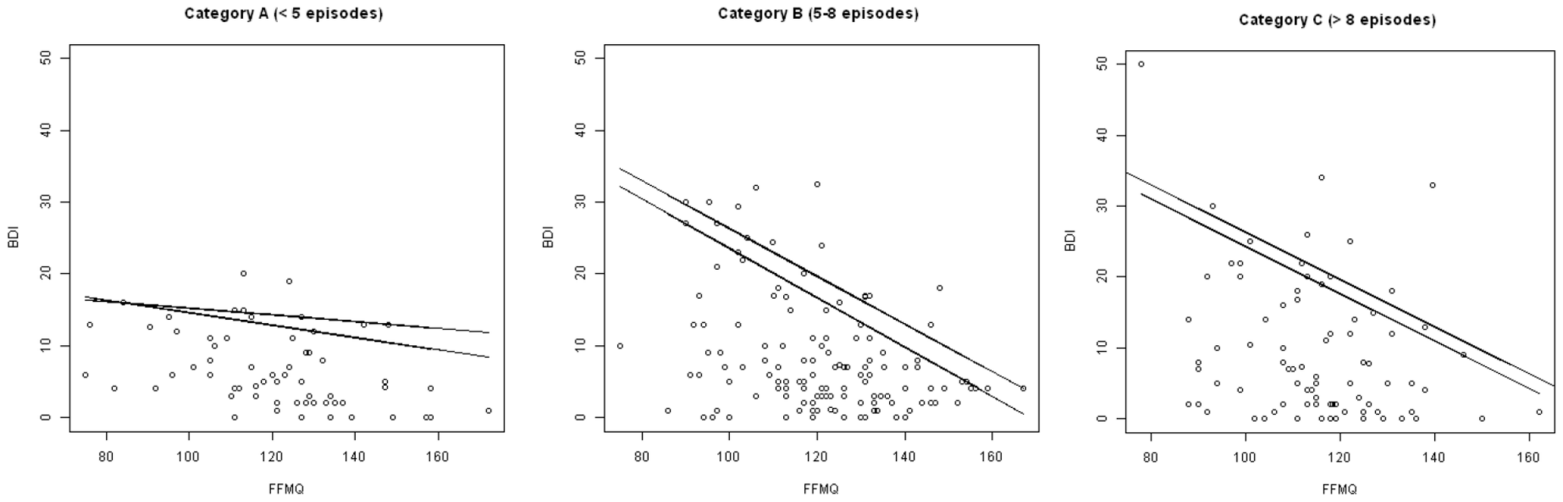
Quantile regression of BDI as a function of FFMQ for subsamples categorised based on number of previous Major Depressive Episodes. Lines represent the slope estimation for the 0.85 and 0.9 quantiles.

Having categorised participants by number of previous episodes, one-way ANOVAs were conducted to examine the influence of previous number of episodes on the relationship between trait mindfulness and residual depressive symptoms. Two models were compared. The first model predicted BDI as a function of FFMQ and previous episode category; and the second model predicted BDI as a function of FFMQ, previous episode category, and an interaction between Category and FFMQ. A significant difference between these models would therefore demonstrate that the interaction based on category is significant. The model was tested at the upper quantiles, which represent the upper boundary of the distribution where FFMQ prove to act as a limiting factor, unbiased by other unmeasured factors which act in the lowest quantiles of the response variable (BDI). Specifically we tested the 75^th^, 80^th^ 85^th^ and 90^th^ quantiles consistent with ecological studies of limiting factors [41], [42]. Results indicated that the models including the interaction were significantly better at predicting BDI at the 85^th^ quantile and 90^th^ quantiles, *F*(2,267) = 5.48, *p*<.01 and *F*(2,267) = 8.72, *p*<.001, respectively, but not at the 75^th^ and 80^th^ quantiles, *F*(2, 267) = 1.141, *p* = 0.32 and *F*(2, 267) = 1.189, *p* = 0.31, respectively. The interaction effect at the highest quantiles resulted from the differences in the relationship between those who had less than five previous episodes of depression and those who had five or more. That is, categories B and C differed significantly from category A, but not from each other, at both the 85th quantile and 90th quantile (see Table 3). This interaction was still significant when we re-ran the analysis, removing the participant with a very high BDI score in category C which may arguably have biased the relationship. The slope estimate at the 90^th^ quantile for the three categories (Figure 2) illustrates the relationship, with steepest slopes observed for categories B and C. This indicates that the limiting effect of trait mindfulness on residual symptoms is more pronounced for those who had five or more previous episodes of depression than for those who had less than five where the level of trait mindfulness is less obviously related to residual symptoms (Figure 2).

**Table 3:** Interaction Based on Previous Number of Episodes Category for FFMQ on BDI-II.

Quantile	Model variables	Estimate	*SE*	*t*	*p*
85	(Intercept)	57.93	6.99	8.28	0.00
	Category A	−34.63	10.54	−3.28	0.00
	Category C	−0.27	19.39	−0.01	0.98
	FFMQ	−0.34	0.05	−6.53	0.00
	Category A×FFMQ	0.25	0.08	2.90	0.00
	Category C×FFMQ	0.01	0.16	0.06	0.94
90	(Intercept)	59.66	6.55	9.10	0.00
	Category A	−39.72	9.74	−4.07	0.00
	Category C	1.63	24.93	0.06	0.94
	FFM	−0.33	0.05	−6.37	0.00
	Category A×FFMQ	0.28	0.08	3.54	0.00
	Category C×FFMQ	−0.01	0.21	−0.07	0.93

*Note*. Intercept = category B. Category A = 3–4 previous episodes, category B = 4–8 episodes, category C = >8 episodes.

### Levels of Mindfulness Required to Limit Residual Depressive Symptoms

The slope estimations of the higher quantiles allowed us to estimate the levels of trait mindfulness that would be required in order to limit residual depressive symptoms, taking into account other potential limiting factors. In order for these estimations to be clinically meaningful we used the cut-offs suggested in the BDI-II manual (minimal <14, mild <20, moderate <29) and used the 85^th^, and 95^th^ quantiles to give a range of associated trait mindfulness scores reflecting the values at which between 85% and 95% of the sample would score under each BDI cut off taking into account all other factors. The FFMQ scores corresponding with minimal depression were 130.29 (85^th^) to 146.00 (95^th^), with mild depression 109.43 (85^th^) to 127.30 (95^th^) and with moderate depression 78.14 (85^th^) to 99.24 (95^th^).

### Testing for Response Bias

In order to ensure that our findings of more pronounced limiting effects in higher quantiles were not simply a result of response bias, i.e. due to individuals reporting greater numbers of previous episodes and using more extreme ends of the scales irrespective of the particular measure, we re-ran analyses substituting the reflection subscale of the Ruminative Response Styles Questionnaire (RSQ) for the FFMQ. The reflection scale indexes adaptive forms of self-related thinking that support successful problem solving and would be expected to have some limiting effect on depressive symptoms, but does not explicitly relate to aspects of decentering and mindfulness that are crucial for reduction of cognitive reactivity. Quantile regression at the upper quantiles .85 and .9 revealed no significant relationship between the RSQ reflection scale and the BDI. The same analyses were conducted but this time with sub-categories based on number of previous episode (as above). Again no significant relationships were found between the RSQ reflection scale and BDI for category A (3–4 episodes), B (5–8 episodes), or C (>8 episodes).

## Discussion

The results of this explorative study support the hypothesis that trait mindfulness acts as a limiting factor for the level of residual depressive symptoms experienced by individuals with a pattern of episodic depression when in remission. The fact that this trend was most pronounced at the upper end of the response distribution suggests that under conditions of lower mindfulness, symptom severity is more free to vary. This could result from a number of unmeasured factors. With high mindfulness, however, symptoms are more constrained regardless of other factors. Furthermore, our results suggest that the limiting effect of mindfulness is most pronounced in patients with higher numbers of previous episodes, for whom residual symptoms are most likely to provide a ground for cognitive reactivity to occur. Although increases beyond 5 previous episodes seemed to show little additional effect. Our findings further suggest that in order to contain residual symptoms at a minimal level under such conditions, relatively high levels of mindfulness would be required, i.e. a trait mindfulness score on the FFMQ of around 130 to 146. Scores of this level have been found to be typical of individuals with a regular meditation practice. For example, Van Dam et al. [43], compared mindfulness scores on the FFMQ in meditators and a non-meditating student sample and found a mean score for meditators of *M* = 144.2, *SD* = 20.9, while the mean score for the student sample without meditation experience, *M* = 126.3, *SD* = 13.8, was slightly below the score predicted to limit depressive symptoms into the minimal range in our study. These findings are in line with results from clinical trials showing that mindfulness interventions exert beneficial effects particularly in patients with higher numbers of previous episodes and unstable remissions [16], and support the notion that intensive training in mindfulness meditation seems necessary to produce such effects. Longitudinal data from the current study showed that FFMQ total scores increased from *M* = 120.3, *SD* = 18.6, at baseline to *M* = 133.0, *SD* = 19.8, at the end of the intervention suggesting that training in MBCT can exert relevant effects in highly vulnerable patients.

Quantile regression allows exploration of relations in contexts where there are potentially many factors that constrain the response variable of the model. Its use in ecological contexts has been predicated on the idea that, under such conditions, the effect of the predictor will be greater in the upper limit of the distribution, where in the case of a significant relationship the response variable will be more uniquely restrained by the predictor, and lower in the lower limit of the distribution, where a number of hidden limiting factors may exert effects. Our findings suggest that this framework provides a fitting way of modelling the relation between residual symptoms and mindfulness. The fact that relations are more pronounced at the upper end of the response distribution for individuals with higher numbers of previous episodes is in line with the theoretical assumption that mindfulness is particularly suited to addressing the problem of cognitive reactivity. Based on the cognitive reactivity or differential activation model [44], individuals who have experienced a greater number of episodes would have more established connections between dysphoric mood and depressogenic thinking patterns, and thus a greater automaticity in the development or worsening of depressive symptoms. Likewise, residual symptoms provide a background from which cognitive reactivity is triggered more easily. From this perspective, our findings are in line with the idea that mindfulness is particularly relevant for those patients in whom reactivity is the dominant issue, while its effects may be less relevant in patients where other and possibly more external factors are of greater relevance, as is often the case for patients with one or two previous episodes. The differential activation account provides a plausible explanation for the observed patterns of findings. However, as the current research did not include any assessments of cognitive reactivity, future research will have to test these assumptions directly. It is important to keep in mind in this context that increased cognitive reactivity may not only occur as a consequence of learning during previous episodes but that it may also reflect pre-existing differences due to genetic and temperamental factors such as neuroticism [45] or effects of adverse events at an early stage of development [46].

Recent studies have highlighted the association between the level of residual symptoms following an episode and risk of future relapse [47], [10]. Our findings are in line with the idea that enhancing levels of mindfulness when highly vulnerable individuals are not in episode is an important target of treatment. The current study suggests a desirable threshold level of mindfulness in terms of constraining residual symptoms for people who have experienced five or more episodes of depression. Monitoring trait mindfulness in these patients, in addition to measures of mood, which are often employed, may provide important insight into individual levels of resilience.

### Limitations and directions for future research

There are a number of limitations to the current study. First, the design was cross sectional and therefore lacks the strength of a longitudinal study, in terms of establishing the causal association between a given level of trait mindfulness and restriction of symptom escalation over inter-episode intervals as well effects on depressive relapse. The current findings however lay the foundation for future work to further examine whether the pattern evident in the current sample can be replicated and extended to longitudinal predictions. Second, the primary outcome and predictor variables for the study were self-report questionnaires, which are vulnerable to response bias. The conceptualisation and measurement of mindfulness is a field that is still developing, and there is some disagreement in the literature as to whether the construct of mindfulness lends itself well to measurement by self-report [48], [49]. However, current findings provide evidence for relations between self-reported mindfulness and more objective behavioural and biological indicators of mindfulness and its consequences and suggest that self-reports provide at least a viable first step into the interrogation of relations between mindfulness and emotional outcomes [50]. A behavioural measure to examine differences in cognitive reactivity as a function of number of previous episodes would have strengthened the conclusions of this study and this is an area for future research. Similarly there is increasing knowledge about the neural substrate and processes underlying vulnerability for depression [51] and the effects of mindfulness [52], which will be useful to take into account for further research. In particular, it has recently been suggested that cognitive reactivity and habitual tendencies towards ruminative thinking arise from increased tendencies towards activation of the default mode network of the brain, a network of brain structures that becomes active during rest and activity of which has been associated with mental processes such as mind wandering and simulation of past and future scenarios [51]. Research in expert meditators has shown that meditation training is associated with a reduced tendency towards activation of this network [53] and it would be interesting to see whether, in clinical populations, levels of trait mindfulness also limit DMN activation at rest.

### Conclusion

In applying an analytical approach that is novel in the field of psychological processes, this study has revealed important relationships between variables, which would not have been evident with standard techniques based around central tendency. The results suggest that naturally occurring trait mindfulness may be an important limiting factor for residual symptoms of depression, and that limiting effects become relevant particularly under conditions of higher residual symptoms and among individuals who have experienced five or more episodes of depression.
